# Quantifying the Spatial Ecology of Wide-Ranging Marine Species in the Gulf of California: Implications for Marine Conservation Planning

**DOI:** 10.1371/journal.pone.0028400

**Published:** 2011-12-06

**Authors:** José Daniel Anadón, Caterina D'Agrosa, Anne Gondor, Leah R. Gerber

**Affiliations:** 1 Ecology, Evolution and Environmental Sciences, School of Life Sciences, Arizona State University, Tempe, Arizona, United States of America; 2 Department of Conservation Biology, Estación Biológica de Doñana, Avda. Américo Vespucio s/n, La Cartuja, Sevilla, Spain; 3 The Nature Conservancy, Tucson, Arizona, United States of America; National Oceanic and Atmospheric Administration/National Marine Fisheries Service/Southwest Fisheries Science Center, United States of America

## Abstract

There is growing interest in systematic establishment of marine protected area (MPA) networks and representative conservation sites. This movement toward networks of no-take zones requires that reserves are deliberately and adequately spaced for connectivity. Here, we test the network functionality of an ecoregional assessment configuration of marine conservation areas by evaluating the habitat protection and connectivity offered to wide-ranging fauna in the Gulf of California (GOC, Mexico). We first use expert opinion to identify representative species of wide-ranging fauna of the GOC. These include leopard grouper, hammerhead sharks, California brown pelicans and green sea turtles. Analyzing habitat models with both structural and functional connectivity indexes, our results indicate that the configuration includes large proportions of biologically important habitat for the four species considered (25–40%), particularly, the best quality habitats (46–57%). Our results also show that connectivity levels offered by the conservation area design for these four species may be similar to connectivity levels offered by the entire Gulf of California, thus indicating that connectivity offered by the areas may resemble natural connectivity. The selected focal species comprise different life histories among marine or marine-related vertebrates and are associated with those habitats holding the most biodiversity values (i.e. coastal habitats); our results thus suggest that the proposed configuration may function as a network for connectivity and may adequately represent the marine megafauna in the GOC, including the potential connectivity among habitat patches. This work highlights the range of approaches that can be used to quantify habitat protection and connectivity for wide-ranging marine species in marine reserve networks.

## Introduction

The Convention on Biological Diversity (CBD) has endorsed the ambitious goal of establishing a comprehensive, effectively managed, and ecologically representative national and regional systems of protected areas globally by 2012 [1]. In marine ecosystems, the number and extent of protected areas has increased recently [2], however existing systems of protected areas are, with few exceptions (e.g., Great Barrier Reef, California coast [3]), neither representative of the world's ecosystems, nor do they adequately address conservation of critical habitat types, biomes and threatened species [4]. Consequently, it is important to develop efficient conservation planning tools to effectively prioritize conservation areas [4].

**Figure 1:**
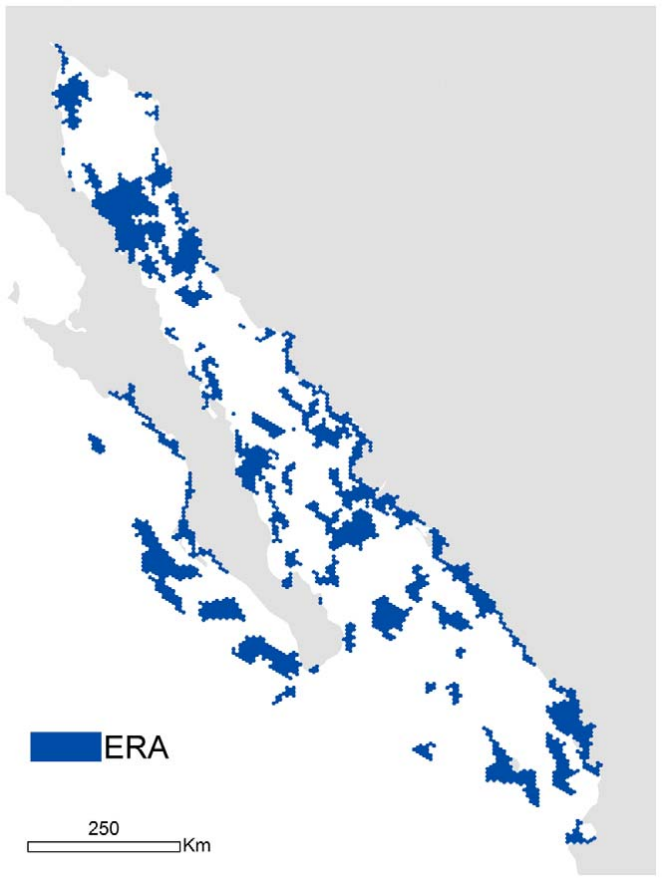
Map of the proposed priority areas for conservation (ERA) in the Gulf of California area.

Due to its high productivity and diversity, the Gulf of California (GOC) is considered a conservation priority area both in Mexico as well as internationally [5]. However, at present, only 7% of the ecoregion is under some form of protection [6]. To reinforce the marine protection in this area, Comunidad and Biodiversidad (COBI) and The Nature Conservancy (TNC) completed a marine ecoregional assessment (ERA) in the Gulf of California (GOC) and near shore Pacific coast of southern Baja California, Mexico (Fig. 1; [7]). This proposal, which combined both species and habitat conservation goals, identified 54 priority areas for conservation covering 26% of the ecoregion (ca. 87,000 km^2^). The aim of this assessment was to identify minimum areas for biodiversity representation and conservation and, in theory, if effective conservation management were implemented in each area, long-term persistence of most biodiversity and productivity in the GOC would be achieved.

The growing movement toward ecosystem-based management and networks of no-take zones requires that they be deliberately and adequately spaced to allow for effective connectivity [8], [9]. Marine Protected Areas (MPAs) are a common biodiversity management and conservation tool, but are often created in an *ad hoc* manner and function independently, even when close to each other. The performance of a network of sites designed with the two-fold purpose of protecting commercial species and allowing for spillover effects will largely depend on the degree to which sites in a network are functionally and structurally linked to each other by both biological (e.g., organism dispersal at different life stages) and physical (e.g., currents) processes [10]. Well-designed networks should include MPAs and other conservation and management areas to support each other by taking advantage of the oceanic currents and movement/migration capabilities of species [e.g.], [ 11]–[13]. Because establishment of isolated marine reserves may not alone suffice for the conservation of biodiversity [9]–[10], [14], testing the level of connectivity between the areas will be a critical aspect in network design [8], [15]. Despite the key importance of connectivity in the design of MPA networks, data on dispersal is sparse for marine organisms [16], [17] and population connectivity in relation to MPAs has only been explored recently [e.g. 13], [18]–[19]. In particular, for the GOC ecoregion, the existing studies have focused only on a portion of the ecoregion, and are primarily geared toward larval dispersal of reef species [18], [20]. Sala et al. [18] proposed that for connectivity between reserves to be meaningful, rocky shore habitat reserves should be spaced evenly across the entire Gulf with a conservative minimum distance of 100 km between sites.

Here, we evaluate the extent to which the proposed ERA configuration of conservation areas in the GOC can function as a network in relation to the habitat protection and connectivity for a number of target species considered as representative of the extant biodiversity. We focus on wide-ranging marine species for two reasons. First, there is increasing awareness for the need of an ecosystem-based management approach to marine conservation. This implies paying particular attention to higher trophic levels, since they play an important role in ecosystem functioning [21]–[23]. In fact, elimination of top predators has led to the degradation of some coastal ecosystems and ecosystem shifts, following changes in the populations of some marine top predators [21]–[25]. Second, the assessment of habitat protection and connectivity at the community level remains a challenge, due to the large differences in life histories and their operating scales among different taxa, particularly in marine ecosystems [16], [26]–[27].

In light of the paucity of spatially explicit data for many marine systems and species, we highlight different approaches and sources of data that can be used to quantify habitat protection and connectivity for wide-ranging marine species. Given the obvious infeasibility of simultaneously monitoring all components of biodiversity at the same time [28], understanding connectivity of a set of carefully chosen individual species may reflect connectivity of the ecosystem [29]–[31]. While the selection of those representative species is not straightforward [e.g. 32], [33], we propose a simple approach based on a multi-criteria expert based approach. The specific goals of the work are: 1) to select representative taxa from the pool of wide-ranging marine species occurring in the GOC, 2) to quantify the habitat protection provided to wide-ranging marine species by the ERA network and 3) to assess the connectivity offered by the ERA to the target species.

## Methods

### Species selection

The selection of the species representative of the wide-ranging biodiversity of the GOC was determined based on expert opinion. In particular, species experts were surveyed within the framework of the *Gulf of California Marine Habitat Connectivity Experts Workshop, June 2007* (see Text S1). The experts were first asked to list the animal species that they considered important for the ecological functioning of the GOC. Note that here we did not constrain our protocol to wide-ranging species, in order to obtain the maximum amount of information from the experts. Then experts rated each species according to five criteria: practicability, socio-economic importance, ecological relevance, spatial scale and conservation status. The contents of the criteria include general conservation considerations, habitat protection and connectivity issues (Table 1). Experts assigned a value from 1 to 5 (1 = least important) for each criterion in the list. Because all respondents did not have information about all species, there is a variable response rate for any given species. For that reason, we averaged the data (within criterion) for each of the five criteria across respondents and then, assuming all criteria were equally important (all weighted 1), we took the grand mean across criteria for each species. To obtain final representative species the ranked list was narrowed by means of three criteria: data availability and representation of the widest possible ranges of taxonomic groups, life history strategies and operating spatial scales.

**Table 1:** Criteria for the selection of representative species of wide-ranging marine species in the Gulf of California.

Criteria	Contain
Practicability	Species likely to respond rapidly to protection
	Species easily identified for monitoring with existing or available technology to enable community involvement.
	Where connectivity occurs in the life cycle: Benthic sessile species that disperse only through larvae. Ontogenetic shifts
	Species with relevant ongoing research (robust baseline data on biology, distribution, etc.
Economic and social relevance	Commercial species
	Relevant for tourism (charismatic megafauna)
	Heritage value
	Recreational and educational value
Ecological Role	Species in different different trophic levels
	Critical ecological roles as apex predators or key trophic links as a adults or juveniles
	Taxonomic, phylogenetic, ecological and/or life history representation
Spatial scale	Represent widest range of connectivity for different spatial scales
	Species that are present in most of the sites of the ERA
Conservation status	Species in some protected status, or identified as conservation targets in protected areas or other efforts
	Invasive species that could be transporting through habitat connections and threatening conservation targets

### Mapping Potential Habitat

The selected species had a comparably large quantity of data. However, for the four species selected available data comprises mainly local studies on habitat use and movement (see Table S1) but no accurate distribution data or enough raw spatial data (i.e. presence/absence) to build classical statistical niche distribution models. For that reason, for the four species, habitat models were built by means of an expert-based modeling approach [34]–[35]. Expert-based models are intended to operate in a data poor environment that precludes the development of empirical based models, and may provide objective and valuable habitat delineation for guiding management efforts [35]–[37]. In our case we develop simple predictive habitat-linkage models based on expert opinion and qualitative models based on the best information available from the literature, ERA database and unpublished data (Table S1). A brief description of the rationale of the modeling procedures for each species is given next. Detailed ecological background and data sources on each species is presented in Text S2 and Table S1. Our study area follows the study area employed in the design of the ERA [7] covering an area of ca. 360,000 km^2^. All GIS work was conducted using ArcInfo GIS 9.1. All resulting grids have a cell size of 200 m.

#### Pelican

We buffered the location of pelican nesting sites to 20 km to represent the maximum distance of pelican movements during the breeding season. Pelicans in the Southern California Bight decreased in abundance with distance from the mainland, but the slope of the regression lines varied considerably [38]. In light of this uncertainty, we assumed a linear decrease in pelican presence and habitat use from the center of the nesting site (high use) to 20 km outward (low use) for each nesting site, and added the resulting grids to reflect the relative overlap and potential intensity of use of the areas surrounding the breeding sites by pelicans to gain additional insight about the location of higher-use areas of breeding pelicans within the ecoregion. Areas with many overlapping nesting grounds would likely contain or be more important to pelicans than one isolated site. We then assigned a value of 4 to the upper half of the range of summed distance values to represent the most important habitat because it has the highest potential overlap of pelicans. The remaining areas of pelican use (i.e., up to 20 km from a nesting site, but with little overlap with other nesting locations) were assigned a value of 3 (high). All other areas were assigned a value of no data/not habitat, assuming pelicans do not fly farther than the 20 km during the breeding season.

#### Hammerhead

Scalloped hammerheads spend daylight hours in shallower waters around seamounts, but move 4–20 km offshore to pelagic areas at night, descending to depths between 50 and 450 m to feed [39]. Scalloped hammerhead distribution within the GOC is also correlated to upwelling events- reportedly leaving the area when cold upwelling water is present, returning shortly after the event is done [40]. Juveniles are mostly coastal, and estuaries in coastal Sinaloa seem to be important nursery grounds [41]. Based on the spatial ecology of the species (see also Supp. Inf.), we modeled potential habitats and assigned them a “quality” or importance value: depth from 0–25 m (medium importance = 2), depth from 26–450 m (lowest = 1), estuaries (high = 3), documented nursery grounds from Villavicencio-Garayzar [41] (highest = 4), seamounts buffered to 20 km (high = 3) and upwelling sites [7] (lowest = 1). We used those seamounts identified in Ulloa et al. [7] as well as those we derived from the analysis of bathymetry using the methods by Kitchingman et al. [42]. The resulting grids were then added to create cumulative habitat types, and recoded such that all cells with a value of 4 or greater were treated as most important habitat (i.e., assigned value of 4). For example, cells with many types of available hammerhead potential habitat have a higher habitat value to hammerheads and a greater potential for hammerhead presence.

#### Leopard grouper

The species is a top-predator from shallow reefs to deep seamounts (>70 m deep) [43]. Adults spawn in aggregations in specific areas within rocky habitats and offshore islands [43]–[44]. To map leopard grouper habitat, we used the following data: documented point locations of grouper [7 and Rupnow et al. unpubl. data], rocky reefs, locations of known grouper spawning aggregations and juvenile settlement areas (19 polygons created for this analysis based on literature review [45 and Sala and Aburto 2000, unpublished WWF technical report data], reef locations for the entire ecoregion derived for this analysis from location of rocky shores and bottom complexity [7], and depth in two classes: 0–30 m and 31–70 m. We then coded these data with individual habitats ranging from lowest (1) to highest (4) importance/quality to grouper (with value of 0 assigned to not important/not habitat) as follows: depths 31–70 m = 1, grouper points and shallow waters = 2, spawning aggregations and rocky reef locations = 4, all else = 0. The resulting grids were then merged to create cumulative habitat types, and recoded such that all cells with a value of 4 or greater are assigned a value of 4. We assumed that cells with many types of available potential grouper habitat have a higher habitat value to grouper, thus a greater potential for grouper presence.

#### Green turtle

The GOC is primarily a foraging area for the green turtle; they prefer areas with seagrasses and shallow waters (<30 m) [e.g. 46]–[48], a habitat frequently found in bays, estuaries and coastal lagoons. When foraging, short and mid-term movements outside core areas (e.g. pacific waters adjacent to coastal lagoons) are performed [47] although short-term movements do not exceed aprox. 20 km [46], [48]–[49]. When migrating into and out of the GOC, sea turtles have a higher likelihood of occurring within 50 km from the shore [50]. We identified key foraging sites for the species, that comprised a) well-known areas important for the species such as Laguna San Ignacio, Bahia Magdalena or Bahia Los Angeles (see Text S1 for a complete list), b) seagrasses locations from the COBI database and c) main estuaries and coastal lagoons in the continental coast of the GOC (e.g. Nayarit, Sonora). Starting from these key sites we identified three scenarios: core areas (key areas plus a 5 km buffer), 5–20 km buffer around key areas, and non-key areas. We overlapped these three scenarios with bathymetry (<30 m) and distance to coast (<50 km) yielding a map with four habitat quality classes (from 1 to 4, Table S2).

### Assessment of habitat protection

Habitat protection was measured as the proportion of the habitat of each species protected by the ERA. Given the differences in the life cycles of the species and their spatial scales, it should be noted that the actual meaning of habitat protection varies among the target species. For hammerhead and grouper, the entire life cycle occurs in the GOC (and potentially in their protected areas), whereas for pelican, protected areas are used for feeding during breeding and green turtle uses the entire GOC as feeding grounds (See Text S2).

### Structural and functional connectivity

Connectivity was assessed by means of metrics related to both structural and functional connectivity. Structural connectivity reflects the physical relationship between habitat patches within the seascape, ignoring the organisms' behavior, whereas functional connectivity attempts to consider how well the seascape facilitates the movement of an organism throughout the seascape and a seascape is more connected if the organism can move freely from patch to patch [51]. As with habitat protection, the actual meaning of connectivity at the GOC scale varies among the considered species. Pelicans were removed from the analysis since likely factors most influencing habitat connectivity for them during breeding season are a trophic primary effect and temporal variations of dynamic features [52] rather than the spatial arrangement of habitat that is evaluated by the connectivity metrics.

Structural connectivity metrics were assessed using FRAGSTATS [53]. We calculated the following metrics (Table 2): number of habitat patches per habitat class (NP), percentage of total seascape area comprised by the largest patch of each habitat class (LPI), mean (and standard deviation of) patch area per habitat class (AREA_Mn and AREA_sd) and mean (and standard deviation) of nearest neighbor distance (EMN_Mn and EMN_sd). Structural connectivity indexes are relative measures of the real connectivity [54] and the relationship between the relative and absolute (real) connectivity values is unknown. To obtain a more biological meaningful measure of connectivity, we compared the structural connectivity of the habitats of the species within the entire GOC against the structural connectivity offered by the habitats included in the ERA sites by means of the ratio M _i,ERA_/M _i,GOC_, where M _i,ERA_ is a given structural connectivity metric of a given species and habitat type in the ERA sites and the whole GOC, respectively. This ratio represents to what extent the ERA captures the natural structural connectivity of the species (as represented by its habitat configuration).

**Table 2:** Metrics calculated employed to quantify the connectivity of the ERA network in the Gulf of California.

Abbreviation	Metric	Definition
NP	Number of patches	Total number of patches of a given type; at seascape level, total number of patches. Measures seascape pattern (or fragmentation).
LPI	Largest patch index	Percentage of total seascape area comprised by the largest patch.
AREA_MN	Mean patch area	Mean patch area across all patches
AREA_SD	Standard deviation of mean patch area	Standard deviation of patch area across all patches
EMN_MN	Euclidean nearest neighbor distance - Mean	Mean nearest neighbor distance across all patches
EMN_SD	Euclidean nearest neighbor distance – Standard deviation	Standard deviation of nearest neighbor distance across all patches
CONNECT	Functional connectivity index	Percentage of the number of functional connections between all patches of the same patch type within a set distance, divided by the total number of possible connections between these patches.Distance thresholds for each species are given in the text

We measured functional connectivity by means of the CONNECT index in FRAGSTATS. This index measures the number of functional links between patches of the same type, where each pair of patches is either connected or not based on a specified distance criterion (i.e. dispersal distance). CONNECT index is reported as a percentage of the maximum possible connectivity given the number of patches. The species' dispersal distance values were used from the literature: 100 km for grouper and 1000 km for hammerhead sharks and the green turtle (see Supp. Inf.). Because of the uncertainty of these dispersal values, in order to assess the robustness of our connectivity metric, we also calculated CONNECT index for dispersal values d/*2* and 2*d, *d* being the average dispersal distance taken from the literature. Following the same rationale as structural connectivity (see above), we compared the functional connectivity of the species in the GOC with the functional connectivity offered by the habitats included in the ERA sites. As before, this ratio represents to what extent the ERA captures the natural functional connectivity of the species.

## Results

### Representative species

Of the 22 experts surveyed, 12 responded (54% response rate). Forty species were considered as relevant for the conservation of the biodiversity and functioning of the GOC (Table S3) and 28 of these species can be considered large vertebrate or wide-ranging species, that constitute the goal of the present work (Table 3). When ranking these 28 species, the top positions included: green turtle (*Chelonia mydas*) and leatherback sea turtle (*Dermochelys coriacea*); hammerhead sharks (*Sphyrna* spp.), sea lion (*Zalophus californianus*); leopard grouper (*Mycteroperca rosacea*); blue whale (*Balaenoptera musculus*), fin whale (*B. physalus*) and sperm whale (*Physeter macrocephalus*) and pelican (*Pelecanos* spp.), yellow-footed gull (*Larus livens*) and osprey (*Pandion haliaetus*). From the top ranked species, data was nonexistent or not available for sea lion (particularly movements in the GOC outside colonies) and for the three species of whales. This filter yielded four taxonomic groups (marine turtles, seabirds, grouper and hammerhead sharks), and from each group we selected the top ranked species. The final result was these four species: a bird species that breeds in the GOC, the California brown pelican (*Pelecanus occidentalis californicus*); a top-predator and wide-ranging fish, the scalloped hammerhead shark (*Sphyrna lewinii*); a more mobility-limited fish, the leopard grouper (*Mycteroperca rosacea*); and a wide-ranging turtle mainly living on seagrasses in the GOC, the green turtle (*Chelonia mydas*).

**Table 3:** List of wide-ranging marine species of the GOC and mean scores for the different criteria obtained from expert-based opinion.

Common Name	Scientific Name	Practicability	Economic & Social relevance	Ecological role	Spatial scale	Conservation status	Mean score
Green turtle*	*Chelonia mydas*	4.67	4.33	3.83	4.50	5.00	4.47
Leopard grouper*	*Mycteroperca rosacea*	4.37	4.63	4.50	4.75	4.00	4.45
Sea lion	*Zalophus californianus*	4.50	3.88	4.38	4.63	3.86	4.25
Hammerhead shark*	*Sphyrna* spp.	3.67	4.33	4.33	4.67	4.17	4.23
Fin whale	*Balaenoptera physalus*	4.00	3.56	3.78	4.89	4.78	4.20
Humpback whale	*Megaptera novaeangliae*	3.67	4.25	3.88	4.25	4.63	4.13
Sperm whale	*Physeter macrocephalus*	4.00	3.57	4.29	4.14	4.57	4.11
Leatherback sea turtle	*Dermochelys coriacea*	3.33	3.83	3.33	4.33	5.00	3.97
Pelicanos*	*Pelecanus* ssp.	4.50	2.88	3.88	4.63	3.75	3.94
Osprey	*Pandion haliaetus*	4.50	2.63	3.75	4.38	4.13	3.88
Yellow footed gull	*Larus livens*	4.25	2.00	4.00	4.75	4.00	3.80
Manta ray	*Manta birostris*	3.50	3.83	3.00	4.00	4.00	3.67
Whale shark	*Rhincodon typus*	3.20	3.40	3.60	3.00	4.80	3.60
Dolphin fish	*Coryphaena hippurus*	3.83	4.17	2.83	4.33	2.67	3.57
Boobie	*Sula* spp.	3.83	2.25	3.38	4.13	4.12	3.54
Mako shark	*Isurus oxyrinchus*	3.00	3.17	3.67	3.67	4.00	3.50
Gulf coney	*Epinephelus acanthistius*	3.00	3.80	3.60	3.80	3.20	3.48
Sierra	*Scomberomorus* spp.	3.67	4.00	3.33	4.00	2.33	3.47
Killer whales	*Orcinus orca*	2.40	3.00	3.57	3.57	4.57	3.42
Frigatebird	*Fregatta magnificens*	3.40	2.29	2.71	4.57	3.60	3.32
Rock scallop	*Spondylus* spp.	3.60	2.80	2.60	3.60	3.75	3.27
Great white shark	*Carcharodon carcharias*	2.14	2.86	3.43	3.86	3.89	3.23
Blue whale	*Balaenoptera musculus*	0.00	4.00	4.00	4.00	4.00	3.20
Olive ridley turtle	*Lepidochelys olivacea*	3.00	2.00	2.00	4.00	3.00	2.80
Tilefish	*Caulolatilus* spp.	2.20	2.75	2.75	3.50	2.50	2.74
Roosterfish	*Nematistius pectoralis*	2.80	2.80	2.60	2.60	2.20	2.60
Hawksbill turtle	*Eretmochelys imbricata*	2.00	2.00	1.00	2.00	5.00	2.40
Loggerhead turtle	*Caretta caretta*	2.00	2.00	1.00	1.00	5.00	2.20

The list only comprises those species considered important for the ecological functioning of the GOC in the Workshop *Gulf of California Marine Habitat Connectivity Experts Workshop, June 2007*.

*indicates the four species finally selected.

### Habitat modeling

**Figure 2:**
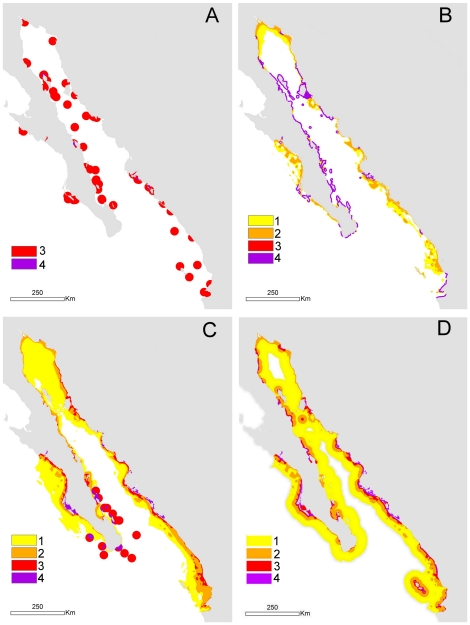
Habitat models for the four representative species in the Gulf of California. Habitat classes for the A) California brown pelican, B) leopard grouper, C) scalloped hammerhead shark and D) green sea turtle.

Our habitat models (Fig. 2 and Table 4) for the GOC, described a similar total area for grouper and pelican habitat (aprox. 50,000 km^2^) whereas the predicted habitat for hammerhead and green turtle was much larger (160,000 km^2^ and 211,000 km^2^ respectively). Figure 2 shows that there is variability across species in terms of where important habitats are found, although overall they were all linked to coastal habitats. Pelican habitat is primarily clustered around islands, whereas grouper, hammerhead and green turtle habitats are closer to shore, but not necessarily island-related. In particular part of the habitat of highest quality for hammerhead and green turtle is linked to estuaries and coastal lagoons.

**Table 4:** Distribution of habitat quality classes in the GOC and the ERA network. Area in km.

Species	Habitat	GOC	ERA	% Included
**Grouper**	0	310585	65523	
	1	23916	6727	28.1
	2	19823	6495	32.8
	3	3	0.5	13.9
	4	17013	8159	48.0
	**All (1–4)**	**50755**	**21382**	**42.1**
**Hammerhead**	0	198054	44270	
	1	97697	19743	20.2
	2	34693	10648	30.7
	3	23985	9067	37.8
	4	6911	3177	46.0
	**All (1–4)**	**163286**	**42635**	**26.1**
**Pelican**	0	307396	68008	
	3	53295	18522	34.8
	4	649	375	57.7
	**All (3–4)**	**53944**	**18897**	**35.0**
**Green Turtle**	0	150183	30313	
	1	146809	35266	24.0
	2	43701	12813	29.3
	3	12345	4125	33.4
	4	8302	4688	56.5
	**All (1–4)**	**211157**	**56892**	**26.9**

### Habitat protection

The amount of habitats represented and potentially protected by the ERA ranges from 24–27% in the case of green turtle and hammerhead respectively to 42% of the habitat of grouper (Table 4). Protection of pelican habitat showed an intermediate value of 35%. For the four species considered, the habitats of higher quality are better represented in the ERA than those of lower quality. The ERA contains around 50% or more of the high-quality habitat for all the four species and the high-quality habitat type is the dominant habitat type for the grouper.

### Connectivity within the ERA

When considering all habitats (1–4) in the GOC, hammerhead and green turtle habitats conform to a single continuous patch whereas for grouper, the largest patch constitutes 36% of all habitats (Table 5). When considering only the best quality habitats (3 and 4), the three species present a naturally fragmented habitat, with the largest patch comprising from 11 to 19% of all habitat. Here hammerheads and green turtles have a much more naturally fragmented habitat than the grouper, as shown by their number of patches and patch size values. The three species exhibit similar habitat fragmentation patterns when considering all habitat classes or only the best habitats.

**Table 5:** Structural and functional connectivity metrics for the habitats of the GOC and the ERA network for the selected species.

Species	Extent	NP	LPI	AREA_Mn	AREA_sd	EMN_Mn	EMN_sd	CONNECT
All habitats (1–4)								
Grouper	GOC	777	36	7819	109594	2110	5889	11.9 (6.0–27.4)
	ERA	638	11	3351	16903	1815	5149	12.7 (6.9–30.3)
Hammerhead	GOC	1	100	16328600	0	-	-	100 (100–100)
	ERA	604	16	7058	38758	1596	5483	97.1 (70.3–100)
Green Turtle	GOC	1	100	21115700	0	-	-	100 (100–100)
	ERA	846	14	6725	42895	972	2828	98.0 (76.9–100)
Best habitats (3–4)								
Grouper	GOC	259	11	6570	21348	4201	11551	14.4 (10.2–25.5)
	ERA	181	12	4508	13329	5968	17184	12.2 (8.5–31.0)
Hammerhead	GOC	756	15	4087	29183	1019	3484	95.9 (65.5–100)
	ERA	647	9	1893	9051	1247	4443	96.9 (68.1–100)
Green Turtle	GOC	776	19	2661	22543	782	2961	99.4 (84.5–100)
	ERA	693	11	1272	6283	1147	6229	99.4 (84.9–100)

Abbreviations of connectivity metrics from Table 4. CONNECT mean value is referred to mean dispersal distance d, where as intervals values indicates CONNECT values with d/2 and d*2 dispersal distances (see Methods). Data in ha.

The differences in habitat configuration between the entire GOC and the ERA, are stronger in those species whose natural habitat conforms to a unique large patch (i.e. hammerhead and green turtle) (Table 6). This pattern also holds, although on a lesser extent, when considering only the best habitats. Hammerheads and green turtles have the largest reduction in mean patch size (46 and 48%) and in the largest patch index (58 and 56% respectively). ERA/GOC ratios on nearest-neighbor distances (EMN) do not match this pattern and the largest increases in EMN are in green turtle and grouper (1.47 and 1.42) (Table 6).

**Table 6:** Structural and functional connectivity ratios ERA/GOC for the selected species.

Species	TYPE	NP	LPI	AREA_m	AREA_sd	EMN_m	EMN_sd	CONNECT
Grouper	1+2+3+4	0.82	0.30	0.43	0.15	0.86	0.87	1.07 (1.11–1.15)
	3+4	0.70	1.03	0.69	0.62	1.42	1.49	0.85 (0.84–0.1.21)
Hammerhead	1+2+3+4*	<0.01	0.16	-	-	-	-	0.97 (0.70–1.00)
	3+4	0.86	0.58	0.46	0.31	1.22	1.28	1.01 (1.04–1.00)
Green turtle	1+2+3+4*	<0.01	0.14	-	-	-	-	0.98 (0.77–1.00)
	3+4	0.89	0.56	0.48	0.28	1.47	2.10	1.00 (1.01–1.00)

For each species all habitats (1+2+3+4) and only the best habitats (3+4) are considered. Abbreviations of connectivity metrics from Table 4. CONNECT mean value is referred to mean dispersal distance d; whereas intervals values indicates CONNECT values with d/2 and d*2 dispersal distances (see Methods).

*indicates that these habitats conform a single continuous patch in the GOC (see Table 5).

Natural functional connectivity (i.e. considering the whole GOC) strongly differed among species, considering all habitats (1–4) or only best quality habitats (3–4). In the latter case, for the whole GOC and for species' average dispersal values, grouper connectivity value was 14%, whereas for hammerhead and green turtles it was 96 and 99% (i.e. almost all patches interconnected among them). Connectivity values considering only those habitat patches included in the ERA were very similar to those in the whole GOC, as reflected by the ratios ERA/GOC, that were very close to 1. These results indicate that connectivity provided by the ERA, as measured by the CONNECT metric, is very similar to the natural connectivity existing in the whole study area. Functional connectivity values varied when considering the uncertainty of the dispersal distance; however, the ratio GOC/ERA was not affected by this source of uncertainty (Tables 5 and 6).

## Discussion

### Representative species

We propose a multi-leveled approach for the selection of representative species, based on a multi-criteria expert approach. The selected representative species comprises three very different taxonomic groups (birds, turtles, fishes), a wide range of positions in the food chain (from herbivorous to apex predator –hammerhead), a wide range of operating spatial scales (that affects the relative importance of the GOC in the life cycle of the species – see Text S1) and a wide range of dispersal distances (approximately from 100 to 1000 km). Thus, the selected species may adequately represent wide-ranging marine biodiversity. Despite their straightforward usefulness for conservation, selecting representative species is not without criticism [33]. Because of the absence of complete knowledge of species' ecologies and their functional roles in ecosystems, the results of our approach should be viewed as hypotheses to test [31]. Our expert-based approach has also yielded two additional results: a list of important species for the ecosystem functioning of the GOC (Table S3) and a ranked list of representative wide-ranging marine species (Table 3). These results are relevant to future conservation and ecological works in the GOC.

### Habitat protection and connectivity

Our results show that the proposed ERA network largely represents the habitats of the four species considered (mean: 30%, range 25–45%). These figures are not surprising given the size of the ERA (26% of the GOC), however highest quality habitats for these four species were better represented (46–57%) than low-quality habitats, indicating very large representation of the best habitats. This range of values of habitat protection for these four species, largely exceeds the goal of 10% for fish, and nearly meets the goal of 40% for birds and turtles, considered in the design of the ERA [7]. As it was intended in the species selection process, the four species represent a wide range of life history strategies among marine or marine-related vertebrates (i.e. bird, reptile, wide-ranging fish and restricted-ranging fish with commercial interest), and thus serve as useful indicators of the vertebrate fauna in the GOC. In any case, it should be noted that we do not know how much habitat is actually needed to assure the viability of the different species. This issue is highly specific [9], [55] and a more detailed analysis addressing the spatial population dynamics with much finer biological information is essential.

Structural fragmentation metrics showed that groupers exhibit a more patchy distribution than the hammerhead and the green turtle in the GOC, likely due to its narrower habitat preferences (i.e. rocky habitats). Similarly, functional connectivity indicates that the populations of species have different connectivity levels (i.e. grouper 10%, green turtle and hammerhead >95%). This metric is a simplification of the real biological system and should be read with caution [53] but, in any case, functional connectivity metrics indicates that very wide-ranging vertebrates (i.e. those with dispersal distances >1000 km) present very high connectivity values (>95%, i.e. 95% of all possible patch-to-patch distance are within dispersal range) when considering the GOC. Despite the above caution, these results suggest that at least these species may be considered to function as a pancmictic population at the scale of the GOC.

Habitat fragmentation in the GOC logically increases when we consider only those areas included in the ERA, as shown by structural metrics and GOC/ERA ratios. Despite this result, the three species' functional connectivity indices (which take into account species-specific dispersal distances) indicate that the level of connectivity among the ERA sites is very similar to the connectivity level considering the whole GOC. Overall, this result is remarkable and indicates that, for the studied species, the ERA may function as a network with natural connectivity levels.

**Figure 3:**
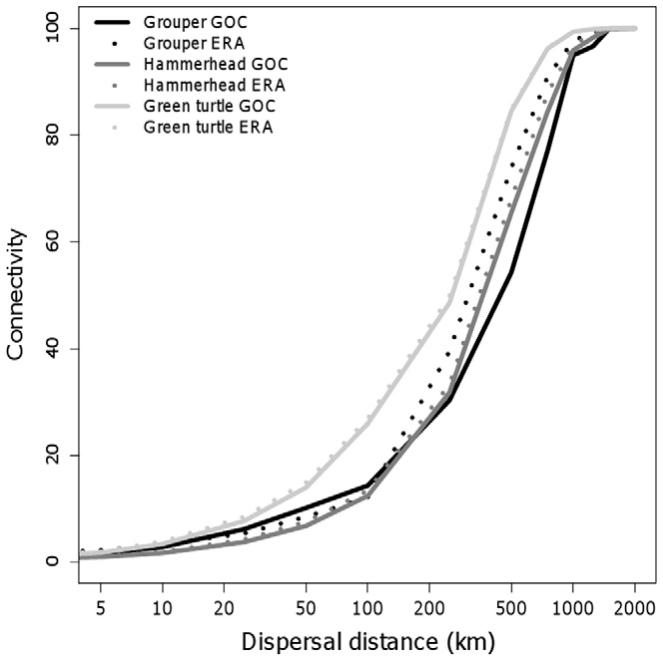
Functional connectivity for the three species habitats considered. Functional connectivity (as measured by the metric CONNECT in FRAGSTATS, see Methods) for different hypothetical dispersal distances and for the three species habitats (grouper, hammerhead and green turtle). Note that most considered dispersal distances are unrealistic for the considered species; dispersal distances described in the literature and employed in this work (see Supporting Information) are 100 km for the grouper and 1000 km for the green turtle and the hammerhead.

Our results for functional connectivity are robust to uncertainty in species dispersal distances. Furthermore, if we plot the hypothetical functional connectivity (as measured by the metric CONNECT) for very different dispersal distances and for the three species (grouper, hammerhead and green turtle), we observe that in all cases they are strikingly similar (Fig. 3). In all cases, connectivity for these three species steadily increases when we assume a dispersal distance of 0 to 100 km; increases steeply with dispersal distances from 100 to 1000 km; and is very high (>95%) at dispersal distances greater than 1000 km. This similarity among species indicates that the inter-species differences in connectivity in the GOC are dependent on dispersal distance and that the distribution of the species' habitat might be of secondary importance. Two possibilities may explain this similarity in the connectivity curves. First, the particular shape of the seascape (peninsula in the middle, long, narrow shape of the GOC) imposes a similar habitat configuration to most species. Second, the considered species all have a coastal life stage and their habitat maps are, to some extent, similar. Species with primarily pelagic, offshore distributions (e.g. mako shark, fin whale) are likely to have a different dispersal distance-connectivity profile. It should be noted here, however, that much of the biodiversity in the GOC uses coastal habitats at various life stages and thus our connectivity profile, common for both GOC and ERA habitats, is likely to be applicable to many of the species in our study area.

### Conservation planning considerations

Our analysis represents a first step in understanding connectivity within and between the ERA network of sites for the Gulf of California. More generally, our work serves to illustrate the complexity of assessing habitat protection and connectivity among marine areas at a community level, with species that disperse at different life stages, but also to provide insight into the beginning of the process (i.e. species selection) and into how one might approach connectivity modeling with different types of data. This work constitutes a first step toward the understanding of habitat protection and connectivity by any eventual MPA network in the GOC. Anyhow, many other species (e.g. algae, invertebrates and small fishes) may clearly depart from our case studies and should be a focus of further research.

Further studies should address the functioning of the target species' populations (i.e. spatially explicit population models) in the context of the ERA network [e.g. 9], [12], [19]. We expect that as we develop more realistic population models they become more species-specific, and thus comprehensive multi-species approaches will become methodologically complex and plagued with large uncertainties [15]. Three aspects are key when considering further efforts to address species population dynamics in relation to MPA networks. First, as shown in this work by the differences between the grouper and the other three species regarding natural functional connectivity, species with different life cycles and operating scales, may perform very differently under the same network conservation system [9]. In this sense, the scale at which a species life cycle, movement and dispersal occurs, is likely to be the most important characteristic that defines the conservation value of a MPA network for a specific species [9], [26]. Second, in the present work we have considered non-protected areas (i.e. matrix) as empty. This is not a biologically realistic assumption and the matrix may play both a positive or negative role [56], [57]. More importantly, this role depends largely on human activities (harvesting, habitat loss and fragmentation, and pollution [5], [7], that can be, at least on paper, the subject of management. More spatially-explicit models, taking into account the role of the matrix, are likely to give information not only on the MPA network, but also on management guidelines of human uses outside the protected areas. Both kinds of information (design and management of protected and unprotected areas) can be, equally valuable for conservation, leading to a more ecosystem-based management (EBM) [58]. Finally, the development of more realistic models relies on high quality species-specific movement, dispersal and population data. Acquiring this kind of information for a large enough number of species to represent the biodiversity present in a given study area is a tremendous challenge and thus inter-institutional and inter-team collaboration and coordination is necessary.

Research on MPA networks and their management has rapidly increased in the last years, leading to the emergence of general frameworks and guidelines [e.g. 9], [10]. Because of the challenge associated with developing realistic spatially-explicit models for particular case studies [8], [12], [19], such “simple rules” as well general methods such as the one developed here are important future research. Finally, it is essential that we begin testing the application of general guidelines that yield coarse quantitative rules of size and spacing [59], both in the GoC and beyond.

## Supporting Information

Text S1 **Life history of the selected representative species.**
(DOCX)

Text S2 **Summary notes of the** ***Marine Habitat Connectivity in the Gulf of California Experts Workshop*** **, 5–6 June 2007 - Tucson, Arizona.**
(DOCX)

Table S1 **Data sets used to model potential habitats for the selected representative species.**
(DOCX)

Table S2 **Construction of the habitat quality classes (1–4 and 0 = non-quality habitat) for the green turtle in the GOC, using distance to key sites, water depth and distance to the coast as criteria (see** **Methods** **section, Text S1 and Table S1 for a description of key sites and further details).**
(DOCX)

Table S3 **List of species considered important for the ecological functioning of the GOC, as assessed from expert-based opinion in the Workshop** ***Gulf of California Marine Habitat Connectivity Experts Workshop, June 2007*** **.**
(DOCX)
