# Supplementary material for: Quantifying the Spatial Ecology of Wide-Ranging Marine Species in the Gulf of California: Implications for Marine Conservation Planning
Source: PLoS One. 2011 Dec 6;6(12):e28400. doi: 10.1371/journal.pone.0028400 (PMC3232197; doi:10.1371/journal.pone.0028400)
Supplement: Text S1 — Life history of the selected representative species. (DOCX) [file pone.0028400.s001.docx]

**SUPPORTING INFORMATION Text S1**

**Life history of the selected representative species**

California brown pelican

California brown pelicans (*Pelecanus occidentalis californicus)* in the ecoregion are thought to be part of a metapopulation that extends from California throughout the GOC and into coastal mainland Mexico [1]. The metapopulation consists of five subpopulations, two (76% of the total metapopulation) are completely contained within the GOC, and an additional two are partially contained in the ecoregion [1]. Approximately 84% of the global population of California brown pelicans breeds on islands in the Gulf of California [1], thus breeding habitat in the GOC is of utmost importance to the survival of this subspecies [1]. Consumption of pesticide-laden fish, lack of food, and disturbances by humans were responsible for a marked decline in reproductive success, and consequently a decline in numbers of brown pelican subspecies in the 1960s and 1970s [2], leading to the species being listed as endangered under the US Endangered Species Act of 1973. Although pelicans are not directly harvested, there are substantial human impacts on the Gulf’s islands (introduced species, human disturbance of nesting grounds). We focused on breeding pelicans for this study because the colonies are well-documented: nesting habitats occur on desert and estuarine islands, and feeding habitats mostly include pelagic, offshore areas within 20 km while breeding [1, 3]. The group scored them high for practicability, spatial scale, ecological importance and conservation status.

Hammerhead shark

Scalloped hammerheads (*Sphyrna lewini)* are a coastal-pelagic, semi-oceanic shark occurring in shallow continental shelf water and adjacent deep water (depth range 0 – 512 m, usually 0 – 25 m), often entering estuaries and other coastal embayments [4-5]. Juveniles and adults (and gravid females) are often found throughout the eastern coast of the GOC [5]. Scalloped hammerheads spend daylight hours in shallower waters around seamounts, but move 4-20 km offshore to pelagic areas at night, descending to depths between 50 and 450 m to feed [6]. Scalloped hammerhead distribution within the GOC is also correlated to upwelling events- reportedly leaving the area when cold upwelling water is present, returning shortly after the event is done [7]. Juveniles are mostly coastal, and estuaries in coastal Sinaloa seem to be important nursery grounds [5]. Neonates in a coastal estuary in Kaneohe Bay, Hawaii reflected a small core habitat-utilization area during the day (average 1.26 ± 1.12 km^2^), followed by more extensive movements at night [8]. However, young hammerheads do not leave the nursery grounds until much older [8]. Based on mtDNA studies of scalloped hammerheads, Duncan et al. [9] conclude that, at a global scale, individual nursery populations may be linked by areas of continuous coastline, and thus have high connectivity, but that oceanic dispersal by females is rare.

Hammerhead sharks in the Atlantic have experienced a decline in abundance of 83% since 1986 [3]. Hammerheads in the ecoregion both migrate poleward in summer with the young, often in large aggregations of hundreds of individuals, and have local populations that remain in one general area year-round [4]. The maximum documented dispersal distance, based on conventional tags, is 1671 km whereas more conservative (smooth) dispersal values are around 1100 km [10], illustrating the species’ ability to disperse widely.

Leopard grouper

Leopard grouper (*Mycteroperca rosacea*), the most abundant and intensely fished grouper in the Gulf, are endemic to the GOC and are distributed throughout the ecoregion [11]. It is a top-predator from shallow reefs to deep seamounts (>70 m deep) [11]. The species exhibits ontogenetic habitat shifts as follows: Adults spawn in aggregations of 150-600 that persist for weeks (early April through June) in specific areas within rocky habitats and offshore islands [11-12]. Spawning aggregations near Loreto occur in areas ranging from 1-20 m deep, throughout the entire water column, in open waters near (2-10 m) the rocky reefs (sand/reef interface) [12]. There is evidence of migration to spawning grounds, since during non-spawning times the abundance of grouper in the surrounding reef is much higher [12]. Spawning is timed so that the larvae can take advantage of abundant food sources in the Central Gulf, when plankton concentrations reach maximum levels [12].

Non-spawning adults have been found in low densities throughout the reefs in areas surrounding the spawning aggregation sites [12] . Non-spawning *M. rosacea* occur year-round in groups. Fish are rarely solitary [12] . Feeding aggregations of >100 individuals form in areas characterized by high densities of small schooling fish (e.g., *Harengula thrissina* and *Cetengraulis mysticetus*) [in 12] .

Larvae persist for a mean of 24.3 (+- 3.6 d) days in the water column [11]. In captivity, larvae must eat a wide variety of heterogeneously- sized foods to obtain ample nutrition [13]. Such diverse plankton naturally occur in the areas chosen as spawning aggregation sites. A few weeks later, when *Sargassum* biomass is at its peak (usually May and June), larvae preferentially recruit to *Sargassum* beds attached to large, shallow (<5m) boulders rather than to bare boulders [11]. Vertical walls and shallow seamounts also have recruiting groupers, but in much lower densities [11]. Post-settled (1.98 cm long) grouper occur between and beneath large boulders after the *Sargassum* sloughed off from July through September [11]. Recruitment is negatively correlated with El Niño years, thus the negative effects of fishing on the population can be exacerbated in an ENSO year, pushing population levels below recovery levels [11]. Sala et al. [14] estimated that reef fish larvae could travel up to 100 km, and Kinlan and Gaines [15] estimated approximately 130 km.

Green turtle

The GOC is mainly a foraging area for the green turtle (*Chelonia mydas)*, and the species migrates into and out of the ecoregion, and also have several juvenile feeding areas in the Midriff Islands region as well as in each of the coastal lagoons along the Pacific Coast of the Baja California Peninsula [16-17]. Green sea turtle foraging habits change according to available food resources. Turtles on the Sonoran coast near Bahia Kino, forage on eelgrass, *Zostera marina*. On the west coast of the Gulf near Bahia de los Angeles, they mainly feed on red algae (*Gracilaria lemaneiformes*), other algae (*Ulva, Codium*) as well as invertebrates, including sea hares (*Aplysia* spp.), several species of sponges, tube worms, and Humboldt squid, *Dosidicus gigas* [16]. On the Pacific coast, green turtles in Bahia Magdalena primarily eat red algae (*G. lemaneiformes, G. pacifica and Hypea johnstonii*) and lesser amounts of eelgrass when inside the bay, and seagrass (*Phyllospadix torreyi*) when outside the bay [1820]. It appears that *Sargassum* is not digestible to them, and they may avoid it [19]. Some black mangrove (*Avicennia germinans*) cotyledons were found in very small amounts in the stomachs of the Bahia Magdalena turtles [18]. Through heavy metal profiling analyses, Talavera-Saenz et al. [20] found that green turtles examined inside Bahia Magdalena are likely foraging outside the bay, possibly in coastal regions with upwelling, supporting the findings of Lopez-Mendilaharsu et al. [18]. Green sea turtles are capable of migrating thousands of kilometers from their nesting grounds to foraging grounds both inside and outside the GOC [16]. It is thought adult females leaving the nesting grounds follow migration corridors within 50 km from shore [16], travelling between 6.18 km/day to 44.7 km/day through satellite telemetry (see references in [16]). In their foraging grounds, the species selects areas with seagrasses and shallow waters (<30m) [e.g. 18, 21-22], a situation frequently found in bays, estuaries and coastal lagoons. When foraging, short and mid-term movements outside core areas (e.g. pacific waters adjacent to coastal lagoons) are performed [18] although short-term movements do not exceed aprox. 20 km [21-23]. Home ranges in Bahia de Los Angeles averaged 409 - 3,908 ha depending on the method used [19]. Diel activity (distance and area within a 24-hr period) ranged from 70 ha to 1252 ha per day, or about 10-52% of their home range [24]. We identified key foraging sites for the species, that comprised a) well known areas important for the species identified in the literature such as Laguna San Ignacio, Bahia Magdalena-Estero Banderitas, El Pardito, Bahia Concepcion, San Bruno-Santa Rosalía, san Rafel- El Barril, Bahia Los Angeles, Isla de San Pedro Martir, and Islas Marias, b) seagrasses locations from the COBI database and c) main estuaries and coastal lagoons in the continental coast of the GOC (e.g. Nayarit, Sonora).

**Supplemental material references:**

[1] Anderson DW, Henny CJ, Godinez-Reyes C, Gress F, Palacios EL, Santos del Prado K, Bredy J (2007) Size of the California Brown Pelican Metapopulation during a Non-El Niño Year: Reston: U.S. Geological Survey, Open-File Report 2007-1299. 35 p.

[2] Anderson DW, Jehl Jr JR, Risebrough RW, Woods Jr, LA, Deweese R, Edgecomb WG (1975) Brown pelicans: improved reproduction off the southern California coast. Science 190: 806 – 808.

[3] Briggs KT, Lewis DB, Tyler WB, Hunt Jr. GL (1981) Brown Pelicans in Southern California: Habitat Use and Environmental Fluctuations. The Condor 83: 1-15.

[4] Baum JK, Myers RA, Kehler DG, Worm B, Harley SJ, Doherty P (2003) Collapse and conservation of shark populations in the northwest Atlantic. Science 299: 389-392.

[5] Villavicencio-Garáyzar CJ (2000) Áreas de crianza de tiburones en el Golfo de California. Universidad Autónoma de Baja California Sur Área Interdisciplinaria de Ciencias del Mar, Informe final SNIBCONABIO proyecto No. L054. México DF.

[6] Klimley AP, Cabrera-Mancilla I, Castillo-Geniz JL (1993) Horizontal and vertical movements of the scalloped hammerhead shark, *Sphyrna lewini,* in the southern Gulf of California, Mexico. Cienc Mar 19: 95-115.

[7] Klimley AP, Butler SB (1988) Immigration and emigration of a pelagic fish assemblage to seamounts in the Gulf of California related to water mass movements using satellite imagery. Mar Ecol Prog Ser 49: 11-20.

[8] Holland KN, Wetherbee BM, Peterson JD, Lowe CG (1993) Movements and distribution of hammerhead shark pups on their natal grounds. Copeia 1993: 495-502.

[9] Duncan, KM, Holland KN (2006) Habitat use, growth rates and dispersal patterns of juvenile scalloped hammerhead sharks *Sphyrna lewini* in a nursery habitat. Mar Ecol Prog Ser 312: 211-221.

[10 ]Kohler NE, Turner PA (2001) Shark tagging: a review of conventional methods and studies. Environ Biol Fish 60: 191–223.

[11] Aburto-Oropeza O, Sala E, Paredes G, Mendoza A, Ballesteros E (2007) Predictability of reef fish recruitment in a highly variable nursery habitat. Ecology 88: 2220-2228.

[12] Erisman BE, Buckhorn ML, Hastings PA (2007) Spawning patterns in the leopard grouper, *Mycteroperca rosacea*, in comparison with other aggregating groupers. Mar Biol 151: 1849-1861.

[13] Gracia-Lopez V, Kiewek-Martinez M, Maldonado-Garcia M, Monsalvo-Spencer P, Portillo-Clark G, Civeda-Cerecedo R, Linares-Aranda M, Robles-Mungaray M, Mazon-Suastegui JM (2005) Larvae and juvenile production of the leopard grouper, *Mycteroperca rosacea* (Streets, 1877). Aquac Res 36: 110-112.

[14] Sala E, Aburto-Oropeza O, Paredes G, Parra I, Barrera JC, Dayton PK (2002) A general model for designing networks of marine reserves. Science 298: 1991-1993.

[15] Kinlan BP, Gaines SD (2003) Propagule dispersal in marine and terrestrial environments: a community perspective. Ecology 84: 2007–2020.

[16] Seminoff JA, Alvarado J, Delgado C, Lopez JL, Hoeffer G (2002) First direct evidence of migration by an East Pacific green seaturtle from Michoacán, México, to a foraging ground on the Sonoran Coast of the Gulf of California. Southwest Nat 47: 314-316.

[17] Koch V et al.(2006) Estimates of sea turtle mortality from poaching and bycatch in Bahía Magdalena, Baja California Sur, Mexico. Biol Cons 128: 327-334.

[18] Lopez-Mendilaharsu M, Gardner SC, Seminoff JA, Riosmena-Rodriguez R (2005) Identifying critical foraging habitats of the green turtle (*Chelonia mydas*) along the Pacific coast of the Baja California peninsula, Mexico. Aquat Conserv: Mar Fresh Ecos 15: 259–269.

[19] Seminoff JA, Resendiz A, Hidalgo S, Nichols WJ (2002) Diet of the East Pacific green turtle, *Chelonia mydas*, in the central Gulf of California, México. J Herpetol 36: 447-453.

[20] Talavera-Saenz A, Gardner SC, Riosmena-Rodriquez R, Acosta Vargas B. (2007) Metal profiles used as environmental markers of Green Turtle (*Chelonia mydas*) foraging resources. Science Total Environ 373: 94 – 102.

[21] Seminoff JA, Resendiz A, Nichols WJ (2002) Home range of the green turtle (*Chelonia mydas*) at a coastal foraging ground in the Gulf of California, México. Mar Ecol Prog Ser 242: 253-265.

[22] Senko J, Lopez-Castro MC, Koch V, Nichiols WJ. 2010. Immature East Pacific Green Turtles (Chelonia mydas) Use Multiple Foraging Areas off the Pacific Coast of Baja California Sur, Mexico: First Evidence from Mark-Recapture Data. Pacific Science 64: 125-130.

[23] Brooks LB, Harvey JT, Nichols WJ (2009) Tidal movements of East Pacific green turtle Chelonia mydas at a foraging area in Baja California Sur, México. Mar. Ecol. Prog. Ser. 386: 263–274.

[24] Seminoff JA, Jones TT (2006) Daily movements and activity ranges of green turtles (*Chelonia mydas*) at a coastal foraging area in the Gulf of California, Mexico. Herp Conserv Biol 1: 81-86.
