# Supplementary material for: Quantifying the Spatial Ecology of Wide-Ranging Marine Species in the Gulf of California: Implications for Marine Conservation Planning
Source: PLoS One. 2011 Dec 6;6(12):e28400. doi: 10.1371/journal.pone.0028400 (PMC3232197; doi:10.1371/journal.pone.0028400)
Supplement: Text S2 — Summary notes of the Marine Habitat Connectivity in the Gulf of California Experts Workshop , 5–6 June 2007 - Tucson, Arizona. (DOCX) [file pone.0028400.s002.docx]

**SUPPORTING INFORMATION Text S2**

**Summary notes of the *Marine Habitat Connectivity in the Gulf of California Experts Workshop, 5-6 June 2007 - Tucson, Arizona***

**Marine Habitat Connectivity in the Gulf of California**

**Experts Workshop, 5-6 June 2007 - Tucson, Arizona**

**SUMMARY**

**I. Introduction**

The World Wildlife Fund (WWF) and The Nature Conservancy (TNC), in 2005 agreed on an action plan to work more collaboratively and strategically at a regional scale in the Gulf of California (the Gulf). The main conservation strategy identified by this collaboration is to design and implement by

2012, a functional network of marine and coastal sustainable management areas (the network) with specific management objectives for each conservation area. As the base for building the network, TNC and WWF Gulf Team have agreed to utilize the 54 conservation areas identified in the Comunidad and Biodiversidad (COBI) and The Nature Conservancy marine ecoregional assessment for the Gulf of California and near shore Pacific coast of southern Baja California (Ulloa, 2006).

The performance of a well-designed network of sites designed with the two-fold purpose of protecting critical and commercial species and allowing for spillover effects will largely depend on whether a) reproduction and replenishment of populations tends to be fairly local, b) larvae are dispersed widely by current patterns, c) adult stages are able to swim between sites, or a combination of these processes. Conservation managed areas would be deliberately and adequately spaced to support each other by taking advantage of the oceanic currents, movement/migration capabilities of species and provide resilience against a range of threats.

The 2006 ecoregional assessment analysis did not specifically emphasize these types of connectivity issues, thus, testing the level of connectivity between the areas will be a critical aspect in the network design. Through the assistance of funding from the TNC -RJKose Granting Program, TNC and WWF convened the Connectivity Experts Workshop to more fully involve Gulf of California scientists and conservation specialists together in designing standardized metrics with which to measure connectivity among the sites of conservation interest in the Gulf.

**II. Participant Presentations**

The workshop began with presentations providing background by NGOs and setting the stage for the connectivity issues. The remainder of the day was dedicated to presentations by participants on the aspects of their research that are relevant to connectivity.

Marianne Kleiberg of TNC, introduced the group to the work TNC is doing with WWF in the Gulf. Ignacio March of TNC, gave background on the priority site selection process by CONABIO and CONANP for Mexico’s National Gap analysis for coastal and marine biodiversity. Alejandro Rodriguez of WWF, presented a brief review of the focus from past to present in the connectivity literature, described some of the threats to the region and emphasized the need for better understanding of the connections between conservation areas. Jorge Torre of Comunidad y Biodiversidad (COBI), described the ecoregional assessment process for the selection of the 54 conservation area sand later gave a talk on the community-based monitoring systems has developed for marine reserves within the Gulf.

**Dr. William Gilly** of Stanford University, presented his work on *Dosidicus gigas*, the Humbolt or Jumbo squid, in the Gulf of California. Major points of emphasis included the impact of squid distribution on both their predators (mammals, large fish, and humans), and their prey during all stages of the squid’s life. Of particular importance is a type of prey fish, myctophids, which are abundant at the edges of the oxygen minimum layer and comprise about 90% of all deep sea fish biomass. An understanding of myctophid distribution and the extent of the oxygen minimum layer could shed light on connectivity within the Gulf. He also made the point that phytoplankton blooms sparked by the run-off of nitrogen fertilizers may be artificially enriching this midwater

environment and thus the range expansion of *D. gigas* into the Gulf.

**Octavio Aburto** of Scripps/Universidad Autónoma de Baja California (UABC) introduced the idea that certain species have different habitat requirements at different stages of ontogenetic development. Specifically his presentation focused on ontogenetic shifts in leopard grouper and snappers where juveniles reside in sargassum and/or sea grass beds, and other coastal environments, like mangroves, before settling on rocky reefs in adulthood. This demonstrates direct connectivity between these disparate habitat types.

**Brad Erisman**, also of Scripps, discussed the importance of both feeding and spawning aggregations. His research also focuses on leopard grouper (and other species of reef fish). Erisman suggested that the current efforts at protection of spawning aggregation sites are not enough and that a greater understanding of feeding aggregation distribution and life history traits (e.g., whether the species are hermaphroditic and the effects of fishing one size class) could have implications to conservation.

**Dr. Steve Gaines** of University of California Santa Barbara related his experiences in the development of the Channel Island Marine Reserve network and the California Marine Life Protection Act Initiative. Of particular interest is the idea of using a conservation area size and spacing model based on the habitat requirements of sedentary species and the dispersal range of mobile species. It would be necessary to gather data on species within the Gulf and also to create a rough model of larval dispersal based on low-resolution ocean current data. Another interesting point was that identifying areas of low connectivity (barriers to connectivity) can add much to the understanding of ecoregional connectivity as well.

**Dr. René Funes** of the Centro Interdisciplinario de Ciencias Marinas (CICIMAR) described his research in mesopelagic larval ecology and explained some of the principles of physical oceanography that he works with, because we lacked an oceanographer. The Gulf shifts dramatically seasonally. It undergoes extreme variations in temperature and current direction. This suggests that sites may be connected via larval transport differently at different times of year. He also illustrated the distribution and possible pathways of dispersal for mesopelagic fish larvae, such as sardines and anchovies.

**Peggy Turk Boyer** of Centro Intercultural para los Desiertos y Océanos, (CEDO) A.C., also highlighted the importance of smaller-scale connectivity in the Upper Gulf. By understanding physical oceanography and seasonal variation in the northern Gulf, they discovered that locally protected areas were actually re-supplying nearby fishing grounds, thus benefiting the local fishers. In addition her presentation addressed the issue of human connectivity in the form of coastal development, and related conservation issues.

**Dr. Leah Gerber** of Arizona State University (ASU) focused on the importance of life history to connectivity. Species may move between sites or habitat types based on their life stage. Reserve networks should be designed to account for these movements or target the most vulnerable life stage of the focal species. Her presentation also highlighted the importance of monitoring population growth in managed areas.

**Dr. Jorge Urban** of UABC discussed his research on the whales of the Gulf. He illustrated the use of using satellite tag data to show how certain species of whale use the protected areas in the Gulf. Dr. Urban also explained that the fin whale population in the Gulf was a unique population, whereas the humpback whale population actually leaves the Gulf at certain seasons. Using diet and genetic studies could shed light on Gulf connectivity.

**Ana Luisa Figueroa** of CONANP spoke of her experience with the Baja to Bering (B2B) Initiative2 , a project designed to foster the creation of a network of MPAs from Baja California Sur to the Bering Sea. Specifically, she talked about the B2B’s method of site selection using score cards that are filled out by a pool of experts. The score cards are used to evaluate the ecologic, biologic, and socio-economic feasibility of protecting specific sites. Ms. Figueroa also suggested that the Gulf of California Ecoregional Marine Reserve Network should take the species selected as significant in the B2B project into account in the site prioritization process.

**Dr. Richard Cudney**, Director of PANGAS, discussed the research being conducted by his multi- institutional collaboration. PANGAS focuses their collaborative research efforts in the Northern Gulf of California in the biological, physical, and social sciences. Project partners strive to answer key questions about how people and the environment interact and how these interactions affect the coastal marine ecosystem. Their aim is to provide scientifically sound information to assist management decisions grounded in the biophysical, social, and political realities of the region.

**Dr. Jorge Torre**, Director of COBI, discussed the community-based monitoring systems that they have developed for 9 marine reserves within the Gulf. He spoke of the 6 steps for establishing community-based fully protected marine reserves. The objectives of their monitoring programs are to evaluate the reserve effect on commercial species abundances, larvae spillover, and ecosystem structure and function.

**III. Criteria to Demonstrate Connectivity**

In order to objectively measure habitat connectivity, criteria of how we want to measure connectivity must be agreed upon. Members of the workshop were asked to deliberate on the criteria to demonstrate connectivity. Each member had an opportunity to offer suggestions and the various suggestions were subsequently organized into five categories:

**1. Oceanographic/ physical connections**

• Ocean currents – Important for modeling passive movement of larvae

• Active larval movement, and ontogeny

• Physical oceanographic features and habitat distribution

• Update coastal map – Current coastal maps outdated and do not accurately represent changes due to development and natural processes

• Species boundaries and areas of low connectivity – Separation between the northern and southern Gulf

**2. Ontogenetic shifts**

• Contiguous areas for connecting life stages – Conservation areas should be large enough and contain enough variation in habitat to cater to the shifting habitat and range demands of certain species as they mature

• Mapping key habitats is essential, particularly the ones currently not in the ERA: e.g., sea grass, sargassum, some bottom habitats.

• Population sources – Sources in the context of a metapopulation source/sink scenario

• Spawning aggregations, and where the spawners originate

**3. Wide-ranging species movement**

• Select species with different dispersal distances and habitat requirements

• Critical habitats for these species are not in use year-round, which could mean a strategy of seasonal restrictions may be appropriate at certain sites, depending on the threat

• Species with specific migration patterns

• Climate change impacts on their movements

**4. Watershed and dispersal of threats**

• Model to simulate stressors from terrestrial sources on the marine environments

• Threats and pollution important to connectivity

• Map where there is run-off

o Develop models to find where the run off pollution is going

o Yaqui valley example – Nitrogen fertilizer run-off causes phytoplankton blooms

• Habitats that are tied to watersheds (e.g. mangrove nursery grounds)

**5. Social Connectivity**

• Documentation/map of distribution of fishing effort, activity, what they are fishing, which community is using what resources in other areas - link to income

• Jurisdictional map, institutional, conservation and governance

• Legal tools mapping: having a spatially explicit database for the legal tools that are available for conservation in the Gulf

• Social networks, communication flows between communities

• Outreach and education projects includes governance and social participation

• Academic networks

• Economic networks

**IV. Criteria for Selection of Species**

In order to model and monitor connectivity within the Gulf, a set of focal species is necessary. The members of the workshop were asked to contribute their thoughts on what criteria should be applied to select focal species that could be representative of connectivity (see below) and also to provide a draft list of prospective species, which included some of the B2B species (Apendix 1, Table 1) as a starting point for the selection process. The criteria for species selection were also deliberated on and member’s suggestions were categorized into the following 5 groups:

**1. Practicality**

• Species likely to respond rapidly to protection

• Species easily identified for monitoring with existing or available technology to enable community involvement.

• Where connectivity occurs in the life cycle

o Benthic sessile species that disperse only through larvae

o Ontogenetic shifts

• Species with relevant ongoing research.

o Species that we have robust baseline data on biology, distribution, etc.

**2. High economic and social importance**

• Commercial species

• Relevant for tourism (charismatic megafuana)

• Heritage value

• Recreational and educational value

**3. Ecological Role**

• Critical ecological roles as apex predators or key trophic links as a adults or juveniles

o Species in different levels of the food chain

• Taxonomic, phylogenetic, ecological and/or life history representation

o Species that may give you different answers with respect to connectivity

• E.g., indicators of link between human terrestrial activity and marine

o Species that are representative of each of the ecological niches

• Dominant, co-dominant, rare and endemic species

o Dominant species could show the function, changes in environment, spawning time and space could be used to help determine big spectra of the ecosystem

**4. Spatial scale**

• Represent widest range of connectivity for different spatial scales

o E.g. species that give insight on entire Gulf

o Species that are present in most of the 54 sites

**5. Conservation status**

• Species in some protected status, or identified as conservation targets in protected areas or other efforts

• Invasive species that could be transporting through habitat connections and threatening conservation targets

**V – Participant List**

| **Participants** | **Institution** | **Expertise** | **E-mail address** |
| --- | --- | --- | --- |
| Octavio Aburto | SCRIPPS | Snappers | [maburto@ucsd.edu](mailto:maburto@ucsd.edu) |
| Brad Erisman | SCRIPPS | Reef fish | [berisman@ieng9.ucsd.edu](mailto:berisman@ieng9.ucsd.edu) |
| Jorge Urbán | UABCS | Cetaceans | [jurban@uabcs.mx](mailto:jurban@uabcs.mx) |
| Ana Luisa Figueroa | CONANP | mpas and community outreach | [afiguero@conanp.gob.mx](mailto:afiguero@conanp.gob.mx) |
| William Gilly | Stanford University | Jumbo squid | [lignje@stanford.edu](mailto:lignje@stanford.edu) |
| Steve Gaines | UC Santa Barbara | Marine reserve design | [gaines@lifesci.ucsb.edu](mailto:gaines@lifesci.ucsb.edu) |
| René Funes Rodríguez | CICIMAR | Ictioplancton | [rfunes@ipn.mx](mailto:rfunes@ipn.mx) |
| Leah Gerber | ASU | monitoring mpas/population analyses | [leah.gerber@asu.edu](mailto:leah.gerber@asu.edu) |
| Peggy Turk Boyer | CEDO | Director CEDO | [peggy@cedointercultural.org](mailto:peggy@cedointercultural.org) |
| Marcia Moreno Baez | UofA/PANGAS | GIS/Remote Sensing | [mamoreno@email.arizona.edu](mailto:mamoreno@email.arizona.edu) |
| Gaspar Soria | UofA/PANGAS | Field testing Northern GOC  circulation models | ? |
| **Core Team** |  | Title |  |
| Caterina D'Agrosa | ASU | Post Doc | [caterina.dagrosa@asu.edu](mailto:caterina.dagrosa@asu.edu) |
| Chris Runcorn | ASU | SoLS Biology Graduate Program | [christopher.runcorn@asu.edu](mailto:christopher.runcorn@asu.edu) |
| Jorge Torre | COBI | Director COBI | [jtorre@cobi.org.mx](mailto:jtorre@cobi.org.mx) |
| Richard Cudney | PANGAS | Director PANGAS | [cud@ag.arizona.edu](mailto:cud@ag.arizona.edu) |
| Anne Gondor | TNC | Conservation Planner | [agondor@tnc.org](mailto:agondor@tnc.org) |
| Marianne Kleiberg | TNC | Program Manager Baja California Sur | [mkleiberg@tnc.org](mailto:mkleiberg@tnc.org) |
| Ignacio March | TNC | Science Director Mexico Program | [imarch@tnc.org](mailto:imarch@tnc.org) |
| Alejandro Rodriguez | WWF | Scientist Gulf of California Office | [arodriguez@wwfmex.org](mailto:arodriguez@wwfmex.org) |
