# Supplementary material for: Quantifying the Spatial Ecology of Wide-Ranging Marine Species in the Gulf of California: Implications for Marine Conservation Planning
Source: PLoS One. 2011 Dec 6;6(12):e28400. doi: 10.1371/journal.pone.0028400 (PMC3232197; doi:10.1371/journal.pone.0028400)
Supplement: Table S1 — Data sets used to model potential habitats for the selected representative species. (DOCX) [file pone.0028400.s003.docx]

**SUPPORTING INFORMATION Table S1**

**Table S1.** Data sets used to model potential habitats for the selected representative species.

| **Species** | **Habitat** | **Source data types** | **Source** |
| --- | --- | --- | --- |
| **Pelicans** |  |  |  |
|  | Rookery locations | Points | Anderson et al. 2007 |
| **Hammerheads** |  |  |  |
|  | Nursery grounds | Points | Villavicencio 2000 |
|  | Estuaries | Polygons | Brusca et al. 2006,  Ulloa et al. 2006 |
|  | Upwelling sites | Polygons | Ulloa et al. 2006 |
|  | Seamounts | Points | Ulloa et al. 2006,  Derived this paper |
| **Leopard grouper** |  |  |  |
|  | Known grouper locations | Points | Ulloa et al. 2006,  J. Rupnow, unpublished data |
|  | Spawning aggregation sites | Polygons | Sala et al. 2003,  Sala and Aburto 2002 |
|  | Rocky shores | Lines | Ulloa et al. 2006 |
|  | Bottom complexity | Polygons | Ulloa et al. 2006 |
| **Green sea turtle** |  |  |  |
|  | Estuaries | Polygons | Brusca et al. 2006,  Ulloa et al. 2006 |
|  | Seagrasses | Points | Ulloa et al. 2006 |
|  | Described key sites | - | Seminoff et al. 2002  Lopez-Mendilaharsu et al. 2005  Koch et al. 2007  Senko et al. 2010 |
