# Supplementary material for: Quantifying the Spatial Ecology of Wide-Ranging Marine Species in the Gulf of California: Implications for Marine Conservation Planning
Source: PLoS One. 2011 Dec 6;6(12):e28400. doi: 10.1371/journal.pone.0028400 (PMC3232197; doi:10.1371/journal.pone.0028400)
Supplement: Table S2 — Construction of the habitat quality classes (1–4 and 0 = non-quality habitat) for the green turtle in the GOC, using distance to key sites, water depth and distance to the coast as criteria (see Methods section, Text S1 and Table S1 for a description of key sites and further details). (DOCX) [file pone.0028400.s004.docx]

**SUPPORTING INFORMATION Table S2**

**Table S2**. Construction of the habitat quality classes (1-4 and 0=non-quality habitat) for the green turtle in the GOC, using distance to key sites, water depth and distance to the coast as criteria (see Methods, Text S1 and Table S1 for a description of key sites and further details).

|  | < 30 m depth | >30 m depth |
| --- | --- | --- |
| Key sites, buffer <5km | 4 | 3 |
| Key sites, buffer 5-20km | 3 | 2 |
| Key sites, buffer > 20km | 2 | If distance to coast <50 km = 1  If distance to coast >50 km = 0 |
