# Supplementary material for: Quantifying the Spatial Ecology of Wide-Ranging Marine Species in the Gulf of California: Implications for Marine Conservation Planning
Source: PLoS One. 2011 Dec 6;6(12):e28400. doi: 10.1371/journal.pone.0028400 (PMC3232197; doi:10.1371/journal.pone.0028400)
Supplement: Table S3 — List of species considered important for the ecological functioning of the GOC, as assessed from expert-based opinion in the Workshop Gulf of California Marine Habitat Connectivity Experts Workshop, June 2007 . (DOCX) [file pone.0028400.s005.docx]

**SUPPORTING INFORMATION Table S3**

**Table S3**. List of species considered important for the ecological functioning of the GOC, as assessed from expert-based opinion in the Workshop *Gulf of* *California Marine Habitat Connectivity Experts Workshop, June 2007.*

| Common Name | Scientific Name |
| --- | --- |
| Hake | *Merluccius productus* |
| Green turtle* | *Chelonia mydas* |
| Leopard grouper* | *Mycteroperca rosacea* |
| Sea lion | *Zalophus californianus* |
| Hammer head shark* | *Sphyrna* spp. |
| Fin whale | *Balaenoptera physalus* |
| Snapers | *Lutjanus* spp. |
| Squid | *Dosidicus gigas* |
| Humpback whale | *Megaptera novaeangliae* |
| Sperm whale | *Physeter macrocephalus* |
| Sardines | *Engraulids/Clupeids spp.* |
| Leatherback sea turtle | *Dermochelys coriacea* |
| Pelicanos* | *Pelecanus occidentalis californicus* |
| Commercial swimming crab | *Callinectes* spp. |
| Osprey | *Pandion haliaetus* |
| Yellow footed gull | *Larus livens* |
| Sea cucumber | *Parasthicopus fuscus* |
| Manta ray | *Manta birostris* |
| Octopus | *Octopus* spp. |
| Commercial species lobsters | *Panulirus* spp. |
| Stingrays | *Dasyatis* spp. |
| Whale shark | *Rhincodon typus* |
| Dolphin fish | *Coryphaena hippurus* |
| Boobie | *Sula* spp. |
| Small groupers | *Palabrax* spp. |
| Curvina | *Cynoscion* spp. |
| Mako shark | *Isurus oxyrinchus* |
| Murex snails | *Murex* spp. *(Hexaplex* spp.*)* |
| Gulf coney | *Epinephelus acanthistius* |
| Sierra | *Scomberomorus* spp. |
| Conch | *Strombus* spp. |
| Killer whales | *Orcinus orca* |
| Sargassum | *Sargassum* spp. |
| Sea urchin | Various species |
| Frigatebird | *Fregatta magnificens* |
| Yellowtail (jurel) | *Seriola lalandi* |
| Rock scallop | *Spondylus* spp. |
| Great white shark | *Carcharodon carcharias* |
| Krill | Various species |
| Pen shells | *Pinna rugosa/Atrina tuberculosa* |
| Blue whale | *Balaenoptera musculus* |
| Oyster catcher | *Haematopus bachmani* |
| Sun star | *Heliaster kubinjii* |
| Clam | *Megapitaria* spp. |
| Canon ball jellyfish | *Stomolophus meleagris* |
| Brown algae | *Padina* spp. |
| Olive ridley turtle | *Lepidochelys olivacea* |
| Mussels | *Mytylidae* |
| Tilefish | *Caulolatilus* spp. |
| Rodoliths | Various species |
| Lanternfish/myctophids | *Myctophidae* |
| Roosterfish | *Nematistius pectoralis* |
| Deadman's fingers (algae) | *Codium* spp. |
| Purpura snail | *Purpura pansa* |
| Hawksbill turtle | *Eretmochelys imbricata* |
| Brittle stars | *Ophiuroidea* |
| Marine isopod (rock lice) | *Ligia* spp. |
| Loggerhead turtle | *Caretta caretta* |
| Tube molluscs | Various species |
| Blue and brown shrimp | Various species |
